# Supplementary material for: Combinatorial Effects of Soluble, Insoluble, and Organic Extracts from Jerusalem Artichokes on Gut Microbiota in Mice
Source: Microorganisms. 2020 Jun 24;8(6):954. doi: 10.3390/microorganisms8060954 (PMC7356569; doi:10.3390/microorganisms8060954)
Supplement: Supplementary file 1 [file microorganisms-08-00954-s001.zip › Supplemental Table.pdf]

Table.S1 PERMANOVA at aim 2

Test statics (p value)	Cont.	A	B	C	B+C
Cont.	-	11.512 (0.012*)	9.168 (0.008**)	0.725 (0.707)	14.110 (0.007**)
A		-	1.386 (0.273)	8.921 (0.015*)	2.878 (0.036*)
B			-	5.772 (0.009**)	1.317 (0.319)
C				-	7.903 (0.012*)
B+C					-

** $p<0.01$, * $p<0.05$; PERMANOVA

Table.S2 PERMANOVA at aim 3

Test statics (*p* value)	Cont.	A	B’	D	E	B’+D	B’+E
Cont.	-	2.064 (0.003**)	3.518 (0.002**)	4.486 (0.002**)	1.598 (0.006**)	4.154 (0.005**)	1.980 (0.001**)
A		-	1.450 (0.041*)	1.705 (0.002**)	1.554 (0.002**)	1.868 (0.007**)	1.269 (0.043*)
B’			-	1.109 (0.081)	2.139 (0.003**)	1.075 (0.203)	1.400 (0.044*)
D				-	3.086 (0.003**)	1.016 (0.232)	2.092 (0.003**)
E					-	2.733 (0.003**)	1.501 (0.006**)
B’+D						-	1.814 (0.008**)
B’+E							-

** $p<0.01$, * $p<0.05$; PERMANOVA
