# Supplementary figures and images for: Combinatorial Effects of Soluble, Insoluble, and Organic Extracts from Jerusalem Artichokes on Gut Microbiota in Mice
Source: Microorganisms. 2020 Jun 24;8(6):954. doi: 10.3390/microorganisms8060954 (PMC7356569; doi:10.3390/microorganisms8060954)

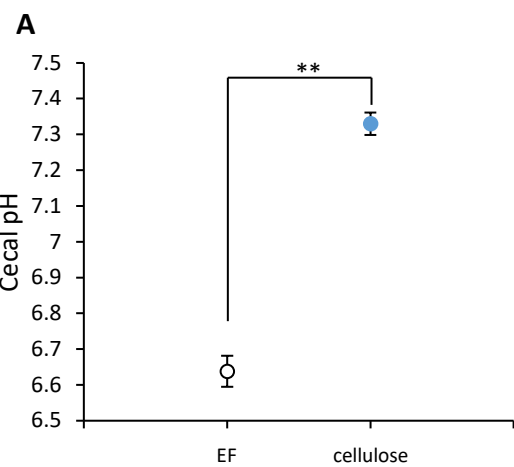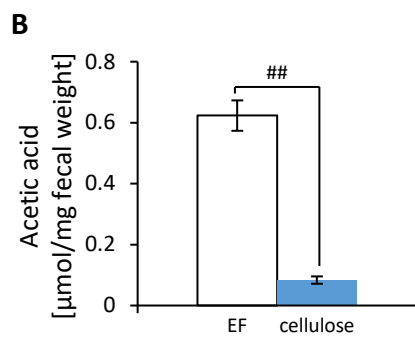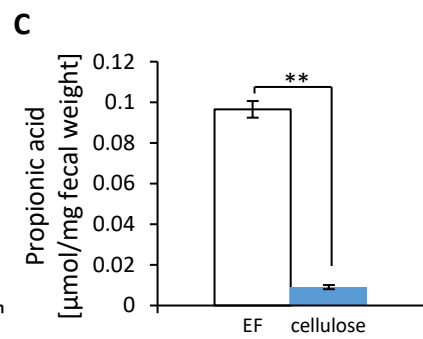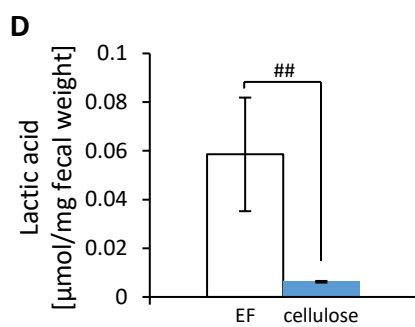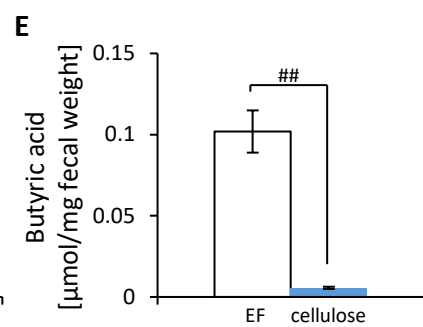

Supplement: Supplementary file 1 [file microorganisms-08-00954-s001.zip › supplemental Figure.pdf]
